# Scholarly Tracks in Emergency Medicine Residency Programs Are Associated with Increased Choice of Academic Career

**DOI:** 10.5811/westjem.2018.1.36753

**Published:** 2018-03-08

**Authors:** Jaime Jordan, Michael Hwang, Amy H. Kaji, Wendy C. Coates

**Affiliations:** * Harbor-UCLA Medical Center, Department of Emergency Medicine, Torrance, California; † David Geffen School of Medicine at University of California Los Angeles, Los Angeles, California; ‡ Los Angeles Biomedical Research Institute at Harbor-UCLA, Torrance, California

## Abstract

**Introduction:**

Career preparation in residency training is not standardized. Scholarly tracks have emerged in emergency medicine (EM) residencies to allow specialized training in an area of focus. The characteristics of these tracks and their value and impact on resident career choice are unknown. We aim to describe the current state of scholarly tracks in residency training programs and their association with pursuit of an academic career.

**Methods:**

Program leaders at EM training programs completed an online survey consisting of multiple-choice items with free-text option. Additionally, participants completed a matrix of dropdown items identifying the immediately chosen post-residency position and applicable track of each member of their graduating class. Descriptive statistics were calculated and reported for multiple-choice items. We performed comparative statistics using chi-squared and Wilcoxon rank-sum tests. Free-text responses were analyzed using a thematic approach.

**Results:**

113/157(72%) programs participated, 51 with and 62 without tracks. Tracks were more common in four-year programs (odds ratio [OR]=4.8;[2.0–11.9]) and larger programs (chi-sq, p=0.001). Perceived benefits of tracks from programs with them included advanced training (46/50; 92%), career guidance (44/50; 88%), mentorship (44/50; 88%), and preparation for an academic career (40/50; 80%). Residents often participated in a single track (37/50; 74%) usually during their later residency years. Programs with tracks were more likely to graduate residents to an academic career, OR 1.8;[1.3–2.4].

**Conclusion:**

This study describes the current characteristics and perceptions of scholarly tracks in EM residencies. Scholarly tracks are associated with an academic position immediately following residency. The results of this study may inform the development and use of scholarly tracks in residency training programs.

## INTRODUCTION

Residency training is designed to prepare residents for careers as practicing physicians who deliver high-quality clinical care to patients. The Accreditation Council for Graduate Medical Education (ACGME) has outlined specific requirements for each specialty training program, including emergency medicine (EM).^1^ However, the ACGME does not provide specific requirements for career preparation, so career preparation is not standardized among EM programs, and residents at different training programs may have different experiences.

It is unknown if we are providing appropriate career guidance and preparation, particularly for careers in academic medicine. Adding to the challenge is the fact that career choice is a complex decision and multiple influential factors play a role, including personal and financial preferences as well as training program characteristics.^2^–^12^ Prior literature has demonstrated that residents may feel ill-prepared for a career in academic medicine due to lack of training, research skills, and mentorship.^2^,^13^ To meet this need, some programs have implemented “scholarly tracks”: longitudinal curricular experiences with clear goals and objectives to allow residents to explore and develop skills in a particular clinical or academic area of focus within EM.^14^ In addition to exposure to a specific area of concentration, tracks may increase scholarly activity, academic success, and selection of a career in academic medicine.^14^–^16^ Despite these potential benefits and suggested strategies for implementation, a recent review of publicly available data demonstrated that specialized tracks are not widespread in EM training programs.^14^,^17^ The reasons for this are unclear. Additionally, the value of specialized tracks and impact on resident career choice remains unknown.

The purpose of this study was to assess the prevalence and characteristics of specialized tracks as well as perceived benefits and barriers to implementation in EM residency training programs. Additionally, we sought to evaluate the relationship between tracks and resident career choice and whether there is an association between tracks and choosing an academic career.

## METHODS

### Study Setting and Participants

We identified ACGME-accredited EM training programs through their accreditation data system.^18^ To prevent duplication, one member of program leadership from each program was invited to participate based on available contact information with preference for seniority (i.e., program director [PD] over assistant/associate program director [APD]). We collected data between March 2017 and June 2017. This study was deemed exempt by the institutional review board of the Los Angeles Biomedical Research Institute at Harbor-UCLA Medical Center.

### Study Design

This was a cross-sectional survey study. We identified contact information for potential participants through the ACGME accreditation data system, Society for Academic Emergency Medicine Residency Directory, Internet search, and personal knowledge of study team members.^18^,^19^ We sent email invitations with a link to an Internet-based survey administered by SurveyMonkey® to potential participants.^20^ Two follow-up emails were sent at bi-weekly intervals to non-responders. Informed consent was implied by those participants who chose to click on the survey link.

### Instrument Development

The instrument was developed by our study group of EM education researchers based on literature review and our prior research in this area according to established guidelines for survey research.^17^,^21^ The survey consisted of multiple-choice items. For items where an “other” choice was available, participants were permitted to enter a free-text response. Participants were asked to complete a matrix of dropdown menus identifying the career choice and track (if present) for each resident in their graduating class. All items were read aloud and discussed among members of the study group to ensure response process validity. We then piloted the survey among a small group of representative subjects, and made revisions based on feedback from pilot testing. The final survey instrument is available in Appendix A. To incorporate all available data and maximize response rate, completion of all survey questions was not required.

**Population Health Research Capsule**

What do we already know about this issue?

Scholarly tracks have emerged in emergency medicine residency training programs, but their value and impact on resident career choice is unknown.

What was the research question?

What is the current state of scholarly tracks? Is there an association between scholarly tracks and pursuit of an academic career?

What was the major finding of the study?

Residency programs with scholarly tracks were more likely to graduate residents to an academic career.

How does this improve population health?

These results may inform the development and usage of scholarly tracks in residency training programs.

### Statistical Analysis

Residency-associated variables included whether tracks were offered, geographic region (West, Southwest, Midwest, Southeast, and Northeast), format (PGY1–3 vs. PGY1–4), total number of residents in the program, number of fellowships offered, and types of fellowships. The tracks were further categorized by whether the tracks were “clinical” (critical care, hyperbarics, pediatric EM, sports medicine, toxicology, ultrasound, wilderness medicine) or “non-clinical” (administration, education, emergency medical services, global health, research, simulation). Resident-level variables included type of track (if the resident came from a program that offered tracks), and the intended educational or employment position after completion of residency. To answer the broad question of whether or not tracks were associated with an academic career, career options were further categorized into academic (academic full-time, academic part-time, fellowship) vs. non-academic (community practice non-teaching, community practice with teaching, other residency, non-clinical career, unknown). Fellowship was included in the academic category as this has been associated with academic career, and fellowship training is strongly recommended by experts in EM as a prelude to an academic career.^7^,^22^,^23^

All data were entered into Microsoft Excel (Microsoft Corporation, Redmond, WA) and transferred to SAS 9.4 (SAS Institute, Cary, NC) for analysis. We calculated and reported descriptive statistics for multiple- choice items. We report the results of comparisons between categorical variables, such as tracks and career choice, using odds ratios and proportions with exact binomial confidence intervals. To compare two cohorts (e.g., tracks vs. those without tracks or academic career vs. non-academic career) with respect to a multi-level categorical predictor (e.g., region), we used the chi-squared test. When comparing continuous variables, such as the number of fellowships offered, we described medians with interquartile ranges and used the non-parametric Wilcoxon rank-sum test. To adjust for potential correlations of residents within residency programs, we used a generalized estimating equation to adjust for clustering by program. Free-text responses were analyzed using a thematic approach.

## RESULTS

### General Results

A total of 113/157 (72%) programs completed the survey. Fifty-one programs reported having tracks. Characteristics of programs with and without specialized tracks are listed in Table 1. There was no significant difference in location between programs with tracks vs. those without (p= 0.6). Tracks were more common in four-year programs (OR = 4.8; [2.0–11.9]) and larger programs (chi-sq, p = 0.001). Programs with tracks were also more likely to offer a greater number of fellowships than those without tracks with medians of 5[2–6] and 3[1–5] respectively; p=0.03. The most common reasons reported for not having tracks was insufficient faculty manpower (28/57;49.1%). Additional reasons are described in Table 2. Written comments from respondents who selected “other” as a reason identified three major themes: 1) the program was in the process of developing tracks; 2) the program or program leadership was new; or 3) an individualized approach to career needs was preferred.

### Description of Tracks

For those programs with tracks, track characteristics are listed in Table 3. Programs had various years of experience with tracks. Track participation was mandatory in 40% (20/50) of programs, usually during the later years in residency. Residents commonly participated in a single track (37/50; 74%) and/or participated continuously (33/50; 66%). Written responses from those selecting “other” for how residents participate in tracks revealed two major themes: residents rotate through all tracks as an intern and then select one in later years, and residents participate in as many tracks as they choose. The most commonly perceived benefits of tracks to residents were an opportunity for advanced training in an area of focus (46/50; 92%), career guidance/exploration/selection (44/50; 88.0%), mentorship (44/50; 88.0%), and preparation for an academic career (40/50; 80.0%) (Table 4).

### Tracks and Careers

Immediate post-residency career is shown in Table 5. Programs with tracks were more likely to graduate residents to an academic position, OR= 1.8 [1.3–2.4]. The type of track pursued, clinical vs. nonclinical was not significantly associated with immediate post-residency academic career, OR = 1.0,[0.6–1.9].

## DISCUSSION

In this study we found that residency programs with tracks were more likely to graduate residents to an academic career. This is not surprising, as tracks offer the opportunity for advanced training, scholarship, and directed mentorship, which have been previously identified as being associated with an academic career.^7^ A four-year program format has also been associated with academic career choice, and in our study we found that tracks were more common in four-year programs.^10^ Interestingly, we did not find an association between type of track completed, clinical vs. nonclinical, and academic career. This was somewhat surprising as one might imagine that residents with an interest and additional training in areas such as administration, education, and research may be more likely to pursue an academic career. However, academicians may have primary job roles that are both non-clinical (i.e., research director, PD) and clinical (i.e. ultrasound director, pediatric EM director).

It is important to note that this study found an association between scholarly tracks and an initial academic position, but this does not necessarily indicate causation. It is not known if the tracks themselves increase the likelihood of choosing an academic career or if the presence of tracks is simply an indicator that a program has more resources and/or specifically encourages academic endeavors as part of its mission. Residents who have a predetermined academic career preference may select training programs with this type of curricular offering to better meet their needs. Some literature demonstrates that residents may not feel well prepared for an academic career.^2^,^13^

In our study population, the majority of residents entered community practice (with and without teaching) immediately following residency. This is similar to what has been reported previously for EM residents.^10^ In contrast to Lubavin’s study in 2004, we found a greater percentage of residents entering fellowships and less an academic career straight after residency.^10^ However, if these categories (fellowships and those who assume an academic position directly after residency) are combined, then our results are similar. Securing an academic position may have become more competitive in recent years, necessitating applicants to gain additional skills and experience. EM leaders strongly recommend fellowship as a precursor to an academic career.^22^,^23^ Fellowship affords protected time to develop expertise in a specific niche without the multiple competing demands of an academic position.

Programs with tracks noted multiple benefits, including advanced training, career guidance, mentorship, and preparation for an academic career. Despite these benefits as well as prior literature suggesting strategies for successful implementation, we found that tracks are not highly prevalent (though there were additional programs in the process of developing tracks).^14^ The most notable reasons for not having tracks in this study were lack of faculty manpower, insufficient time, and lack of administrative resources. These barriers may explain why tracks were more common in larger programs and those with a four-year format as these programs may have a larger faculty to share the workload, greater administrative resources, and more time and flexibility to incorporate such curricula. As there is scant literature defining and reporting objective outcomes resulting from implementing tracks, programs may also be hesitant to devote resources to their development and implementation until further research is done.

For those programs with tracks, our study found that residents usually participate in one track continuously in their later years, with some offering an exploratory rotation through tracks in the earlier years. This likely is by design to meet the overall objectives of such curricula. Trainees need time to consider and select an area of focus that most interests them. Concentrating on a single area with focused mentorship facilitates the development of specialized expertise, allowing for consistent growth and accomplishment of scholarly work.

## LIMITATIONS

This was a survey study and the results are subject to the limitations inherent to this type of data collection. As this was a cross-sectional study, only one period of time was evaluated and it is possible that results may vary if multiple years were incorporated, longitudinally. Additionally, data were collected from only one member of the residency leadership team. This may have led to limited insight in the free-response section, and confirmation of accuracy of individual data was not available. Although the survey response rate was 72%, since we do not have information on the non-respondents, there may have been selection bias.

Additionally, not all respondents completed every survey item, and thus, we may have missed some information. Despite these limitations, we feel this study provides important information regarding scholarly tracks. Our results suggest there is an association between programs with scholarly tracks and selection of an academic career. Furthermore, many perceive benefits of tracks. There are still many questions left unanswered, and research should focus on defining objective outcomes from implementing tracks and whether the association between tracks and the selection of an academic career is due to the tracks themselves or the self-selection of residents.

## CONCLUSION

This study describes the current prevalence, characteristics, and perceived benefits of scholarly tracks in residency training. Scholarly tracks are associated with an academic position immediately following residency. The results of this study may inform the development and usage of scholarly tracks in residency programs.

## Supplementary Information



## Figures and Tables

**Table 1:** Comparison of characteristics between residency programs with and without tracks.

	Programs without tracks (n= 62)*	Programs with tracks (n= 51)*
Region		
West	11/57 (19.3%)	8/50 (16.0%)
Southwest	5/57 (8.8%)	4/50 (8.0%)
Midwest	16/57 (28.1%)	12/50 (24.0%)
Southeast	13/57 (22.8%)	8/50 (16.0%)
Northeast	12/57 (21.1%)	18/50 (36.0%)
Program format		
PGY-1–3	48/57 (84.2%)	28/50 (56%)
PGY-1–4	9/57 (15.8%	22/50 (44.0%)
Other	0/57 (0%)	0/50 (0%)
Total number of residents		
15 or less	0/57 (0%)	0/50 (0%)
16–30	23/57 (40.4%)	7/50 (14.0%)
31–45	22/57 (38.6%)	18/50 (36.0%)
46–60	8/57 (14.0%)	16/50 (320%)
61 or more	4/57 (7.0%)	9/50 (9.0%)
Number of fellowships		
Median, [interquartile range]	3 [1–5]	5 [2–6]
Fellowships currently offered		
Administration	18/57 (31.6%)	22/50 (44.0%)
Critical care	8/57 (14.0%)	14/50 (28.0%)
Education	14/57 (24.6%)	21/50 (42.0%)
EMS	22/57 (38.6%)	26/50 (52.0%)
Global health	16/57 (28.1%)	17/50 (34.0%)
Hyperbarics	2/57 (3.5%)	0/50 (0%)
Pediatrics	18/57 (31.6%)	20/50 (40.0%)
Research	15/57 (26.3%)	23/50 (46.0%)
Simulation	12/57 (21.1%)	14/50 (28.0%)
Sports medicine	7/57 (12.3%)	12/50 (24.0%)
Toxicology	10/57 (17.5%)	14/50 (28.0%)
Ultrasound	35/57 (61.4%)	37/50 (74.0%)
Wilderness medicine	6/57 (10.5%)	5/50 (10.0%)
None	9/57 (15.8%)	6/50 (12.0%)
Other	6/57 (10.5%)	7/50 (14.0%)

*EMS*, emergency medical services; *PGY*, post-graduate year.

* 6 Participants, 5 from programs without tracks and 1 from a program with tracks, answered the question about the presence of tracks, but did not complete any additional questions in the survey.

**Table 2:** Reasons residency programs do not have tracks.

	n (%) Total n = 57
We don’t have the faculty manpower to support tracks	28 (49.1%)
There is insufficient time in the resident schedule	19 (33.3%)
We don’t have administrative resources to support tracks	16 (28.1%)
We do not feel that tracks would be helpful	15 (26.3%)
Our residents don’t want tracks	15 (26.3%)
Other	14 (24.6%)
There is inadequate funding to support tracks	12 (21.1%)
We don’t have leadership support for tracks	8 (14.0%)
We don’t have enough faculty expertise to offer tracks	7 (12.3%)
We don’t know how to implement a track program	5 (8.8%)

**Table 3:** Characteristics of residency program tracks

	Response rate (%)
Length of time program has had tracks	
Less than 1 year	9/50 (18.0%)
1–3 years	14/50 (28.0%)
4–6 years	15/50 (30.0%)
7 or more years	12/50 (24.0%)
Track participation is mandatory	
Yes	20/50 (40.0%)
No	30/50 (60.0%)
Years that residents participate in tracks	
PGY-1	26/50 (52.0%)
PGY-2	41/50 (82.0%)
PGY-3	48/50 (96%)
PGY-4	22/50 (44.0%)
Other	2/50 (4.0%)
Total time residents engage in tracks during residency	
1–4 weeks	2/50 (4.0%)
5–8 weeks	2/50 (4.0%)
9–12 weeks	6/50 (12.0%)
13–16 weeks	3/50 (6.0%)
More than 16 weeks	0/50 (0%)
Continuously	33/50 (66%)
Other	4/50 (8.0%)
Track participation format	
Residents rotate through all available tracks	0/50 (0%)
Residents rotate through multiple tracks	1/50 (0%)
Residents select one track to participate in	37/50 (74%)
Other	12/50 (24.0%)
Tracks offered	
Administration	34/50 (68.0%)
Critical care	21/50 (42.0%)
Education	39/50 (78.0%)
EMS	36/50 (72.0%)
Global health	29/50 (58.0%)
Hyperbarics	2/50 (4.0%)
Pediatrics	14/50 (28.0%)
Research	27/50 (54.0%)
Simulation	22/50 (44.0%)
Sports medicine	12/50 (24.0%)
Toxicology	24/50 (48.0%)
Ultrasound	40/50 (80.0%)
Wilderness medicine	19/50 (38.0%)
Other	12/50 (24.0%)

*EMS*, emergency medical services; *PGY*, post-graduate year.

**Table 4:** Perceived benefits of tracks.

	n (%) Total n = 50
Advanced training in an area of focus	46 (92.0%)
Career guidance/exploration/selection	44 (88.0%)
Directed mentorship	44 (88.0%)
Development of a niche	42 (84.0%)
Preparation for an academic career	40 (80.0%)
Preparation for a leadership role	32 (64.0%)
Creation of a collaborative network	25 (50.0%)
Increased scholarly productivity	25 (50.0%)
Improved wellness during residency	17 (34.0%)
Improved clinical skills	7(14.0%)
Other	3 (6.0%)
None	0 (0%)

**Table 5:** Immediate post-residency career.

Career category	Immediate post-residency career	Residents from programs without tracks n (%), total n= 517	Residents from programs with tracks n (%), total n= 267	All residents n (%), total n= 784
Academic				
	Academic- full time	33 (6.4%)	26 (9.7%)	59 (7.5%)
	Academic- part time	22 (4.3%)	7 (2.6%)	29 (3.7%)
	Fellowship	95 (18.4%)	82 (30.7%)	177 (22.6%)
Non-academic				
	Community practice non-teaching	271 (52.4%)	108 (40.4%)	379 (48.3%)
	Community practice with teaching	78 (15.1%)	41 (15.4%)	119 (15.2%)
	Other residency	1 (0.2%)	0 (0%)	1 (0.1%)
	Non-clinical practice	1 (0.2%)	0 (0%)	1 (0.1%)
	Unknown	16 (3.1%)	3 (1.1%)	19 (2.4%)
