# Supplementary material for: Scholarly Tracks in Emergency Medicine Residency Programs Are Associated with Increased Choice of Academic Career
Source: West J Emerg Med. 2018 Mar 8;19(3):593–9. doi: 10.5811/westjem.2018.1.36753 (PMC5942030; doi:10.5811/westjem.2018.1.36753)
Supplement: Supplementary file 1 [file wjem-19-593-s001.pdf]

Specialized Tracks

We want to learn more about the presence, structure, and outcomes of specialized tracks or "selectives" in emergency medicine training programs. We consider a track to be an organized, longitudinal curricular component for residents to explore and/or gain experience in a specialized area or niche of emergency medicine.

* 1. Does your residency program have specialized tracks?

- ○ Yes
- ○ No

Specialized Tracks

* 2. Please indicate reasons why your program does not offer specialized tracks. (Please select all that apply.)

- ☐ Do not feel that they would be helpful
- ☐ Our residents don't want tracks
- ☐ There is insufficient time in the resident schedule
- ☐ We do not have the faculty manpower to support tracks
- ☐ We do not have enough faculty expertise to offer tracks
- ☐ We do not have leadership support for tracks
- ☐ We don't have administrative resources to support tracks
- ☐ There is inadequate funding to support tracks
- ☐ We don't know how to implement a track program
- ☐ Other (please describe other reasons or offer additional comments to clarify your choices above)

* 3. Please select the region where your residency program is located.

- ○ **WEST**
  (AK, CA, CO, HI, ID, MT, NV, OR, UT, WA, WY)
- ○ **SOUTHWEST**
  (AZ, NM, OK, TX)
- ○ **MIDWEST**
  (IA, IL, IN, KS, MI, MN, MO, NE. ND, OH, SD, WI)
- ○ **SOUTHEAST**
  (AL, AR, DC, DE, FL, GA, KY, LA, MD, MS, NC, SC, TN, VA, WV)
- ○ **NORTHEAST**
  (CT, ME, MA, NH, NJ, NY, PA, RI, VT)

* 4. What is your program format?

- ◯ PGY 1-3
- ◯ PGY 1-4
- ◯ Other (please specify)

* 5. How many total residents are in your program (including interns)?

- ◯ 15 or less
- ◯ 16-30
- ◯ 31-45
- ◯ 46-60
- ◯ 61 or more

* 6. What fellowships does your department currently offer? (Please check all that apply.)

- [ ] Administration
- [ ] Critical Care
- [ ] Education
- [ ] EMS
- [ ] Global Health
- [ ] Hyperbarics
- [ ] Pediatrics
- [ ] Research
- [ ] Simulation
- [ ] Sports Medicine
- [ ] Toxicology
- [ ] Ultrasound
- [ ] Wilderness Medicine
- [ ] None
- [ ] Other (please specify)

Specialized Tracks

7. Please complete the following table with the corresponding career choice of each resident in your 2017 graduating class:
If a resident plans to pursue multiple career options, please select their* ***primary *career choice.***

	Career Choice
Resident 1	
Resident 2	
Resident 3	
Resident 4	
Resident 5	
Resident 6	
Resident 7	
Resident 8	
Resident 9	
Resident 10	
Resident 11	
Resident 12	
Resident 13	
Resident 14	
Resident 15	
Resident 16	
Resident 17	
Resident 18	
Resident 19	
Resident 20	

Other (If you selected "Other" in one or more of the categories above please describe)

Specialized Tracks

* 8. How long has your program had tracks?

- ○ Less than 1 year
- ○ 1-3 years
- ○ 4-6 years
- ○ 7 or more years

* 9. Is participation in a track(s) mandatory?

- ○ Yes
- ○ No

* 10. Please indicate the years in which residents participate in tracks. (Please select all that apply.)

- ☐ PGY-1
- ☐ PGY-2
- ☐ PGY-3
- ☐ PGY-4
- ☐ Other (please specify)

* 11. Please indicate the total amount of time residents engage in their track(s) during residency.

- ○ 1-4 weeks
- ○ 5-8 weeks
- ○ 9-12 weeks
- ○ 13-16 weeks
- ○ More than 16 weeks
- ○ Continuously
- ○ Other (please describe)

* 12. Please indicate how your residents participate in tracks.

- ○ Residents rotate through ALL available tracks
- ○ Residents rotate through multiple tracks
- ○ Residents select one track to participate in
- ○ Other (please specify)

* 13. What tracks does your program offer?

- [ ] Administration
- [ ] Critical Care
- [ ] Education
- [ ] EMS
- [ ] Global Health
- [ ] Hyperbarics
- [ ] Pediatrics
- [ ] Research
- [ ] Simulation
- [ ] Sports Medicine
- [ ] Toxicology
- [ ] Ultrasound
- [ ] Wilderness Medicine
- [ ] Other (please describe)

* 14. What do you believe are the benefits of tracks to your residents?

- [ ] Advanced training in an area of focus
- [ ] Career guidance/exploration/selection
- [ ] Creation of a collaborative network
- [ ] Development of a niche
- [ ] Directed mentorship
- [ ] Improved clinical skills
- [ ] Improved wellness during residency
- [ ] Increased scholarly productivity
- [ ] Preparation for a leadership role
- [ ] Preparation for an academic career
- [ ] None
- [ ] Other (please describe)

* 15. Please select the region where your residency program is located.

- ◯ **WEST**
  (AK, CA, CO, HI, ID, MT, NV, OR, UT, WA, WY)
- ◯ **SOUTHWEST**
  (AZ, NM, OK, TX)
- ◯ **MIDWEST**
  (IA, IL, IN, KS, MI, MN, MO, NE. ND, OH, SD, WI)
- ◯ **SOUTHEAST**
  (AL, AR, DC, DE, FL, GA, KY, LA, MD, MS, NC, SC, TN, VA, WV)
- ◯ **NORTHEAST**
  (CT, ME, MA, NH, NJ, NY, PA, RI, VT)

* 16. What is your program format?

- ◯ PGY 1-3
- ◯ PGY 1-4
- ◯ Other (please specify)

* 17. How many total residents are in your program (including interns)?

- ◯ 15 or less
- ◯ 16-30
- ◯ 31-45
- ◯ 46-60
- ◯ 61 or more

* 18. What fellowships does your department currently offer? *(Please check all that apply.)*

- [ ] Administration
- [ ] Critical Care
- [ ] Education
- [ ] EMS
- [ ] Global Health
- [ ] Hyperbarics
- [ ] Pediatrics
- [ ] Research
- [ ] Simulation
- [ ] Sports Medicine
- [ ] Toxicology
- [ ] Ultrasound
- [ ] Wilderness Medicine
- [ ] None
- [ ] Other (please specify)

Specialized Tracks

19. Please complete the following table with the corresponding track and career choice of each resident in your 2017 graduating class:

If a resident spent time in multiple (but not all) tracks , please select the track of primary focus

If a resident plans to pursue multiple career options, please select their primary career choice

	Track	Career Choice
Resident 1		
Resident 2		
Resident 3		
Resident 4		
Resident 5		
Resident 6		
Resident 7		
Resident 8		
Resident 9		
Resident 10		
Resident 11		
Resident 12		
Resident 13		
Resident 14		
Resident 15		
Resident 16		
Resident 17		
Resident 18		
Resident 19		
Resident 20		

Other (If you selected "Other" in one or more of the categories above please describe)
